# Alterações da Rigidez Arterial em Pacientes com Estenose Aórtica Grave Submetidos à Cirurgia de Troca Valvar

**DOI:** 10.36660/abc.20190577

**Published:** 2021-03-03

**Authors:** Renata Raimundo, Francisca Saraiva, Raquel Moreira, Soraia Moreira, Ana Filipa Ferreira, Rui J. Cerqueira, Mario Jorge Amorim, Paulo Pinho, António Sousa Barros, André P. Lourenço, Adelino Leite-Moreira

**Affiliations:** 1 University of Porto Faculty of Medicine Department of Surgery and Physiology Porto Portugal Cardiovascular Research and Development Center, Department of Surgery and Physiology, Faculty of Medicine of the University of Porto, Porto - Portugal; 2 Centro Hospitalar Universitário São João Department of Cardiothoracic Surgery Porto Portugal Department of Cardiothoracic Surgery, Centro Hospitalar Universitário São João, Porto - Portugal; 3 Centro Hospitalar Universitário São João Department of Anesthesiology Porto Portugal Department of Anesthesiology, Centro Hospitalar Universitário São João, Porto - Portugal

**Keywords:** Valva Aórtica/cirurgia, Estenose da Valva Aórtica/cirurgia, Substituição da Valva Aórtica/métodos, Análise de Onda de Pulso

## Abstract

**Fundamento:**

Pouco se sabe sobre o impacto da estenose aórtica (EA) grave na rigidez aórtica e se ocorre alguma alteração após a remoção da barreira de EA com a cirurgia de substituição da válvula aórtica (SVA).

**Objetivo:**

Estimar as mudanças na velocidade de onda de pulso carotídeo-femoral (VOP) após a cirurgia de SVA e definir os preditores de VOP alta em pacientes com EA grave.

**Métodos:**

Estudo de coorte retrospectivo unicêntrico, incluindo pacientes com EA grave submetidos à cirurgia de SVA com bioprótese, entre fevereiro de 2017 e janeiro de 2019, e medições da VOP (Complior®) antes e depois do procedimento (2±1 meses). Antes e depois da SVA, os valores da VOP foram comparados por meio de testes pareados. foram analisadas as associações de VOP com dados clínicos, bem como aplicados modelos de regressão linear multivariada para estimar os preditores independentes da VOP pré- e pós-operatória. O nível de significância foi estabelecido em 5%.

**Resultados:**

Foram incluídos na amostra 150 pacientes, com média de idade de 72±8 anos, sendo 51% deles do sexo masculino. Identificamos um aumento estatisticamente significativo nos valores de VOP após a cirurgia (9,0 ± 2,1 m/s vs. 9,9 ± 2,2, p<0,001, antes e depois da SVA, respectivamente) e uma associação inversa com as variáveis de gravidade da EA. No modelo de regressão linear multivariada, idade e pressão arterial sistólica (PAS) foram estabelecidas como preditores independentes da VOP pré- e pós-operatória mais alta, enquanto o gradiente valvar médio mais alto foi considerado um determinante da VOP pré-SVA mais baixa.

**Conclusão:**

Identificamos uma correlação inversa da rigidez arterial com a gravidade da EA em pacientes acometidos, e um aumento significativo nos valores da VOP após a cirurgia de SVA. Idade avançada e PAS elevada foram associadas a valores mais altos da VOP, embora as medidas de função arterial estivessem dentro da normalidade. (Arq Bras Cardiol. 2021; 116(3):475-476)

## Introdução

A estenose aórtica degenerativa (EA) é a valvulopatia cardíaca (VPC) mais prevalente em países desenvolvidos e a mais comumente adquirida no mundo, sendo moderada ou grave em 5% dos pacientes com mais de 75 anos nos EUA.^1^ O tratamento padrão-ouro para sua forma sintomática grave é a substituição da válvula aórtica (SVA).^2^^–^^4^

O processo degenerativo da válvula aórtica (VA), que resulta em EA, apresenta alterações fisiopatológicas semelhantes ao processo aterosclerótico responsável pelo aumento da rigidez arterial.^5^ A comunidade científica, portanto, é levada a postular que, na EA, algum grau de disfunção vascular e, consequentemente, rigidez arterial também podem estar presentes.^6^

O envelhecimento também contribui para o enrijecimento vascular, aumentando a pressão aórtica. A medida da velocidade de onda de pulso aórtica (VOP) é o método padrão-ouro, não invasivo e mais reprodutível para avaliar a rigidez arterial.^7^ Basicamente, avalia a capacidade de recuo elástico da aorta, que é diminuída em uma aorta mais rígida, traduzindo-se em um valor de VOP mais alto.^8^^–^^10^ Portanto, em um paciente com EA, espera-se uma VOP pré-operatória mais elevada, que pode ser recuperada após a SVA. No entanto, alguns estudos têm mostrado que essa associação pode não ser linear,^11^ ou seja, mesmo após o procedimento que alivia a obstrução valvar, uma medida de VOP elevada pode ser observada (ou até maior do que a da avaliação pré-intervenção), representando um aumento na carga vascular.^12^

Há uma forte associação entre uma VOP mais alta e hipertensão sistólica, bem como outros fatores de risco cardiovascular (CV) e doença aterosclerótica.^13^^,^^14^ A VOP também tem sido sugerida para prever eventos CV fatais e não fatais, como acidente vascular cerebral ou síndromes aórticas e coronárias.^8^^,^^9^^,^^15^^,^^16^

Há evidências conflitantes em relação às alterações da função arterial após SVA e se a VOP pode ser um marcador de gravidade de EA ou não. Tendo isso em vista, este estudo teve como objetivo esclarecer essa associação.

## Objetivo

O objetivo principal deste estudo foi avaliar a rigidez arterial antes e após a cirurgia de SVA em pacientes com EA grave, usando um equipamento de medição da VOP. Também objetivamos identificar os preditores de resultados da VOP pré- e pós-operatórias nesses pacientes.

## Métodos

### Desenho do Estudo e Pacientes

Estudo retrospectivo unicêntrico, incluindo 150 pacientes com EA grave submetidos à cirurgia SVA com biopróteses entre fevereiro de 2017 e janeiro de 2019, com medições de VOP pré- e pós-operatórias. Pacientes com regurgitação aórtica moderada ou grave concomitante ou procedimentos múltiplos foram excluídos.

### Coleta de Dados e Variáveis

Dados pré-operatórios, cirúrgicos e pós-operatórios foram coletados de prontuários médicos e bancos de dados. Em relação às variáveis pré-operatórias, além dos valores de VOP, também coletamos dados sobre pressão arterial, informações demográficas, fatores de risco cardiovascular, terapia médica em andamento, estado funcional, sintomas e ecocardiograma transtorácico. Cross-clamp (XCT) e tempo de circulação extracorpórea (TCEC), etiologia da doença valvar aórtica e tipo de prótese foram as principais variáveis cirúrgicas. As variáveis de seguimento foram os resultados do ecocardiograma transtorácico (média de 3,9±1,6 meses de seguimento) e a avaliação da VOP.

O comitê de ética local aprovou o estudo, e todos os dados foram anônimos para análise.

### Medição da VOP

Um método não invasivo (Complior^®^ Analyze) foi usado para avaliar a rigidez arterial por meio da VOP carotídeo-femoral antes e depois da cirurgia de SVA. Após alguns minutos em posição supina de repouso, para estabilizar a frequência cardíaca e a pressão arterial, a pressão arterial foi avaliada por meio de um esfigmomanômetro padrão. A distância carótido-femoral foi medida com fita métrica e os dados foram digitados em software específico. Os sensores femoral e carotídeo foram mantidos até que o software atingisse linhas estabilizadas e a melhor qualidade de sinal (acima de 90%). Cada paciente foi submetido a pelo menos duas medições de VOP em cada sessão. Essas medições foram feitas na admissão dos pacientes no Departamento de Cirurgia Cardiotorácica, no dia anterior ou no mesmo dia da cirurgia. A avaliação pós-operatória ocorreu em média 2,2±1,4 meses após a cirurgia.

### Análise Estatística

A distribuição de dados contínuos foi verificada por meio da análise visual dos histogramas e confirmada pelo teste de Shapiro-Wilk. Variáveis contínuas são apresentadas como média e desvio-padrão. As variáveis categóricas foram expressadas em frequências absolutas e relativas. O teste t de amostras pareadas foi usado para comparar variáveis contínuas em dois momentos distintos. As correlações entre a VOP e outras variáveis contínuas foram avaliadas por meio do teste de correlação de Pearson. Os valores de VOP foram comparados entre os grupos usando o teste t independente. Um modelo de regressão linear multivariada foi construído para estimar os preditores da VOP com base em variáveis clínicas relevantes para a avaliação da rigidez arterial. Os pressupostos da regressão foram verificados, os resíduos foram normalmente distribuídos e as variáveis independentes não foram altamente correlacionadas. Foi utilizado nível de significância de 5%. As análises estatísticas foram realizadas no Statistical Package for the Social Sciences (SPSS) versão 24 e no ambiente de linguagem R versão 3.6 (R Core Team - 2018. R: Uma linguagem e ambiente para computação estatística. R Foundation for Statistical Computing, Viena, Áustria. URL https://www.R-project.org/. Frank E Harrell Jr - 2019. rms: Estratégias de modelagem de regressão. Pacote R versão 5.1-3.1).

## Resultados

### Amostra

A caracterização da amostra está representada na Tabela 1. A idade média dos sujeitos foi 72±8 anos, sendo 51% deles do sexo masculino. Hipertensão arterial estava presente em 125 pacientes (83%), dislipidemia em 114 (76%), diabetes em 52 (35%) e histórico de tabagismo em 36 (24%). Quarenta (27%) pacientes foram admitidos com classe funcional ≥ III da New York Heart Association (NYHA). A maioria dos indivíduos (91%) fazia uso de medicamentos anti-hipertensivos na admissão.

**Tabela 1:** Características de base

Variável	n=150
Idade, em anos, média (DP)	72,5 (7,6)
Sexo masculino, n (%)	77 (51,3)
NYHA ≥ III, n (%)	40 (26,9)
Hipertensão, n (%)	125 (83,3)
Atualmente em uso de medicamentos anti-hipertensivos (%)	136 (90,7)
Diabetes, n (%)	52 (34,9)
Dislipidemia, n (%)	114 (76,0)
Histórico de tabagismo, n (%)	36 (24,0)
Índice de massa corpórea, kg/m^2^, média (DP)	28,6 (4,3)
Obesidade (IMC ≥30.00 kg/m²), n (%)	55 (36,7)
Doença arterial coronária, n (%)	19 (12,7)
Arteriopatia extracardíaca, n (%)	22 (14,7)
Doença renal crônica (DC <85ml/min), n (%)	87 (58,0)
Depuração de creatinina (DC), ml/min, média (DP)	83,7 (29,7)

IMC: índice de massa corpórea; min: minuto; NYHA: New York Heart Association; DP: desvio-padrão. Arteriopatia extracardíaca foi considerada se o paciente tinha claudicação, oclusão carotídea ou estenose >50%, amputação devido a doença arterial, intervenção anterior ou planejada na aorta abdominal, artérias dos membros ou carótidas, ou histórico de acidente vascular cerebral. A doença arterial coronariana foi definida quando os pacientes haviam sido submetidos a intervenção coronária percutânea no passado, ou tinham estenose coronariana >50%, mas sem indicação de cirurgia.

Durante a cirurgia, 12% dos pacientes tiveram etiologia congênita confirmada (Tabela 2).

**Tabela 2:** Dados pré-operatórios

Variável	n=150
**Etiologia da válvula aórtica, n (%)**	
Degenerativa	132 (88,0)
Congênita	18 (12,0)
Tempo de CEC, minutos, média (DP)	78 (26)
Tempo de *clamp* aórtico, minutos, média (DP)	57 (20)

CEC: circulação extracorpórea; DP: desvio-padrão.

### VOP Pré- e Pós-operatória

**Figura 1:**
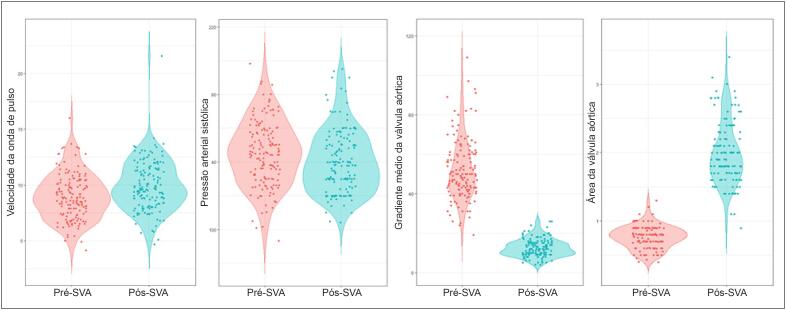
Gráficos em violino dos valores pré- (vermelho) e pós-operatório (azul) da velocidade da onda de pulso (A), pressão arterial sistólica (B), gradiente médio da válvula aórtica (C) e área da válvula aórtica (D).

Observamos um aumento significativo nos valores de VOP pós-operatória, variando de 9,0±2,1 m/s a 9,9±2,2 m/s após a cirurgia de SVA (p<0,001, Figura 1A).

### Dados de Monitoramento

Os valores de pressão arterial sistólica (PAS), gradiente médio da válvula aórtica (GMV) e área da válvula aórtica (AVA) antes e depois da cirurgia de SVA estão representados na Figura 1 (Painéis B, C e D, respectivamente).

### Associações da VOP

**Figura 2:**
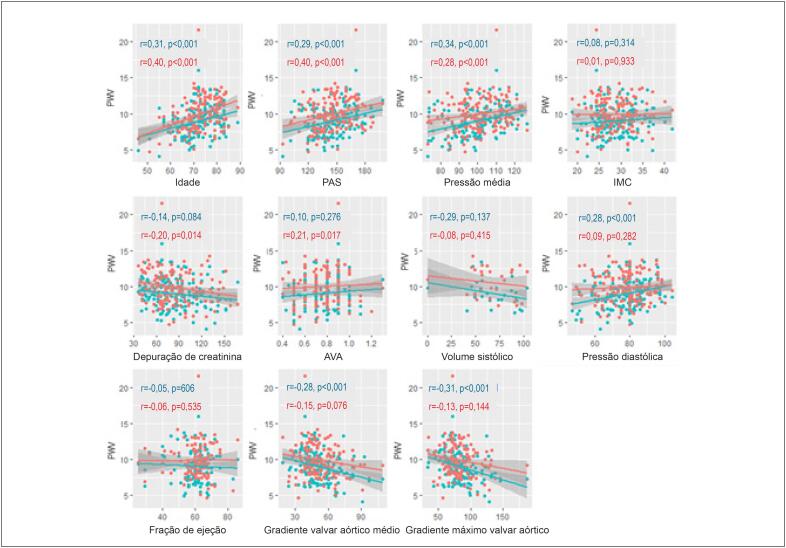
Gráficos de dispersão das relações pré-operatórias (azul) e pós-operatórias (vermelho) da VOP com idade, PAS: pressão arterial sistólica, pressão média; IMC: índice de massa corpórea, depuração de creatinina; AVA: área da válvula aórtica, volume sistólico, pressão diastólica, fração de ejeção, gradiente valvar aórtico médio e gradiente máximo valvar aórtico.

A Figura 2 mostra a análise univariada considerando associações da VOP com potenciais preditores. A VOP pré- e pós-operatória teve correlações positivas com idade, PAS e pressão arterial média (PAM), mas foi inversamente associada com as variáveis de gravidade da estenose aórtica. Não encontramos diferenças na VOP de acordo com gênero, hipertensão arterial, diabetes, tabagismo ou VA bicúspide.

**Figura 3:**
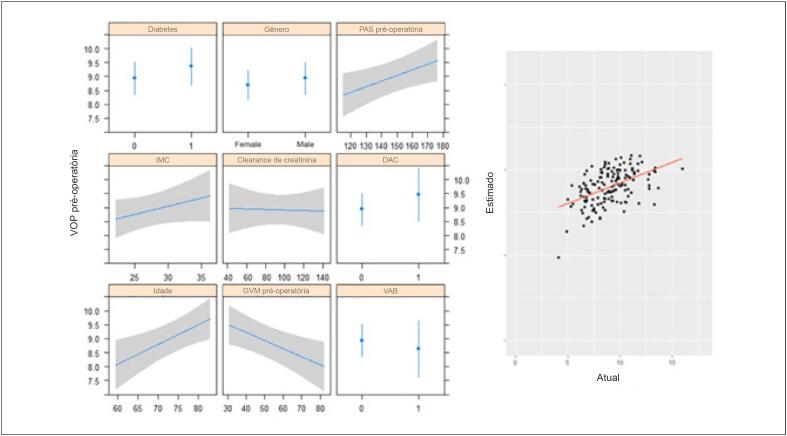
Modelo de regressão linear multivariada de VOP pré-operatória e valores estimados versus reais. VAB: válvula aórtica bicúspide; DAC: doença arterial coronariana; GVM: gradiente médio da válvula; PAS: pressão arterial sistólica; IMC: índice de massa corpórea.

**Figura 4:**
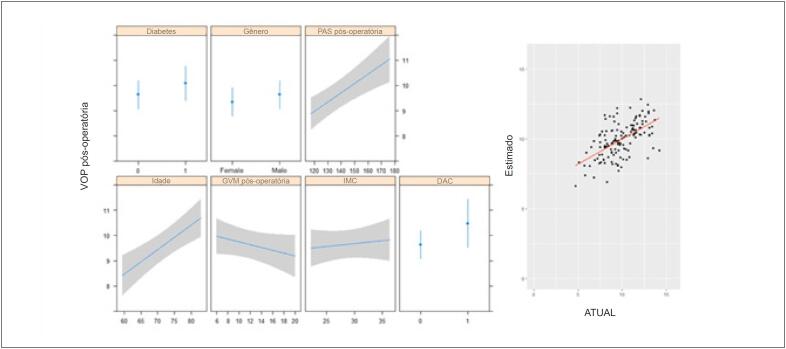
Modelo de regressão linear multivariada da VOP pós-operatória e valores estimados versus reais. IMC: índice de massa corpórea; DAC: doença arterial coronariana; GVM: gradiente médio da válvula; PAS: pressão arterial sistólica.

No modelo de regressão linear multivariada, idade e PAS foram preditores independentes de VOP pré-operatória mais alta, enquanto o GVM mais alto foi preditor de VOP pré-operatória mais baixa (Tabela 3 e Figura 3). Idade e PAS foram considerados preditores independentes de VOP pós-operatória mais alta (Tabela 4 e Figura 4).

**Tabela 3:** Resumo da análise de regressão multivariada (variável dependente: VOP pré-operatória)

Variável	β	EP	b	*p*
Intercepção	0,593	2,576	0,23	0,818
Pressão arterial sistólica	0,020	0,008	2,42	0,017
Diabetes	0,428	0,344	1,24	0,215
Depuração de creatinina	-0,001	0,007	-0,14	0,889
Doença arterial coronária	0,531	0,498	1,07	0,288
IMC	0,059	0,040	1,46	0,147
Idade	0,070	0,025	2,87	0,004
Sexo	0,244	0,336	0,73	0,469
GVM	-0,029	0,011	-2,75	0,007
Válvula aórtica bicúspide	-0,299	0,498	-0,60	0,549

IMC: índice de massa corpórea; β: coeficiente de regressão não padronizado; b: coeficiente padronizado; GVM: gradiente médio da válvula; EP: erro padrão do coeficiente.

**Tabela 4:** Resumo da análise de regressão multivariada (variável dependente: VOP pós-operatória)

Variável	β	EP	b	p
Intercepção	-2,706	2,391	-1,13	0,260
Pressão arterial sistólica	0,037	0,009	4,11	<0,001
Diabetes	0,448	0,352	1,27	0,205
Doença arterial coronária	0,837	0,508	1,65	0,102
IMC	0,023	0,040	0,58	0,564
Idade	0,097	0,023	4,13	<0,001
Sexo	0,287	0,351	0,82	0,415
GVM	-0,056	0,038	-1,46	0,146

IMC: índice de massa corpórea; β: coeficiente de regressão não padronizado; b: coeficiente padronizado; GVM: gradiente médio da válvula; EP: erro padrão do coeficiente.

## Discussão

Este estudo retrospectivo mostrou uma correlação inversa da rigidez arterial com a gravidade da EA e um aumento significativo nos valores de VOP após a cirurgia de SVA em pacientes com EA grave. Idade avançada e PAS elevada foram associadas a valores mais altos de VOP, embora as medidas de função arterial estivessem dentro da normalidade.

A VOP reflete o enrijecimento arterial devido à perda das propriedades elásticas funcionais da aorta. Trata-se de uma das primeiras manifestações de dano estrutural reversível à parede do vaso, e a VOP é considerada uma técnica com aplicabilidade clínica tanto para identificar quanto para estratificar doenças cardiovasculares.^8^^,^^16^

Avaliar o impacto da EA degenerativa na árvore arterial permanece um desafio, uma vez que os mecanismos subjacentes à interação da função vascular e valvar continuam quase desconhecidos.^17^

Liu, et al.^5^ mostraram associação do aumento da VOP com maior escore de cálcio na VA.^5^ Esses resultados sugerem que a deposição de cálcio é uma via importante no processo degenerativo da válvula e da parede aórtica. Além disso, mostraram associação entre aumento da VOP e gradiente de pressão AV avaliado por ecocardiografia. Alterações fisiopatológicas semelhantes são provavelmente compartilhadas entre a degeneração da VA e o enrijecimento das grandes artérias. Korkmaz et al.,^18^ estimaram o enrijecimento arterial usando o Índice Tornozelo-Braquial (ITB) e encontrou maior rigidez arterial em pacientes com esclerose da válvula aórtica,^18^ sugerindo que a degeneração da VA e o enrijecimento das grandes artérias poderiam ter alterações fisiopatológicas semelhantes. De fato, Emir Cantuk et al.,^11^ relataram uma associação significativa entre a gravidade da EA e o aumento da VOP.^11^ Por outro lado, nosso estudo mostra uma correlação inversa entre os gradientes da VA e a VOP pré-operatória. Isso pode ser parcialmente explicado pela obstrução que pode influenciar nas medidas das propriedades arteriais, mascarando os reais efeitos da carga vascular na aorta. El-Chilali et al,^6^ que estudaram pacientes mais velhos (>70 anos) com EA grave e mediram a VOP de forma invasiva, observaram o mesmo: uma correlação inversa entre GVM e VOP.^6^ Essa hipótese é reforçada pelo aumento da VOP observado após a cirurgia de SVA.

Existe um número limitado de estudos que avaliaram a função vascular aórtica em pacientes com EA grave após a intervenção até o momento.^11^^,^^19^^,^^20^ Canturk et al.,^11^ não encontraram diferenças significativas na VOP após a cirurgia.^11^ Por outro lado, Nemes et al..^20^ demonstraram melhora da função vascular um ano após a SVA.^20^ Em nosso estudo, a VOP aumentou significativamente após a cirurgia de SVA. Alguns mecanismos são considerados para explicar esse resultado: o alívio da obstrução valvar após a SVA leva a árvore arterial a operar em um nível de pressão mais elevado, aumentando a carga vascular. Inclusive, Yotti et al.,^19^ mostraram que o alívio da obstrução do fluxo de saída aumenta imediatamente as pressões arteriais e a impedância vascular, induzindo um comportamento vascular mais rígido.^19^ De maneira geral, esses achados refletem-se na prática clínica, uma vez que é comum iniciar tratamento com anti-hipertensivos após a correção da EA, seja por implante de válvula aórtica transcateter (TAVI) ou por cirurgia.^19^^,^^21^^,^^22^

Outra explicação, apontada por Barbetseas,et al.,^12^ seria que o aumento da rigidez arterial após a cirurgia de SVA ocorre devido à lesão da parede aórtica e destruição de *vasa vasorum*, bem como alteração da composição das fibras da parede aórtica, resultando em enrijecimento aórtico. No entanto, devemos reforçar que essa diminuição da distensibilidade aórtica foi avaliada uma semana após a SVA, enquanto seis meses após a cirurgia, a função aórtica melhorou, atingindo níveis semelhantes aos pré-operatórios.^12^ Isso pode representar um efeito transitório descrito pelos autores como “atordoamento da raiz aórtica”.^23^

Um estudo que comparou TAVI e SVA mostrou alterações significativas na VOP apenas em pacientes cirúrgicos, sugerindo que, em pacientes submetidos à TAVI, as propriedades elásticas são mantidas porque não há manipulação cirúrgica.^24^

Em nosso estudo, a VOP também apresentou associação significativa com a idade, que foi considerada um preditor independente de valores mais elevados de VOP. A alteração da VOP em idosos já está bem estabelecida na literatura.^8^^,^^17^^,^^25^ Estudos mostram um aumento da VOP com o envelhecimento, provavelmente relacionado à dilatação e enrijecimento da aorta, uma vez que a impedância aumenta e a complacência arterial diminui com o envelhecimento.^8^^,^^26^ Nosso estudo dá suporte a esses resultados, sendo que idade foi fortemente correlacionada com a VOP pré- e pós-operatória.

A hipertensão arterial sistêmica também se mostrou um preditor de aumento da VOP,^9^^,^^27^ o que está de acordo com nossos resultados, uma vez que PAS elevada foi associada a à VOP elevada. Como mais de 80% da nossa amostra tinha hipertensão sistêmica, não encontramos diferenças substanciais entre os pacientes com e sem esse fator de risco, mas reconhecemos que a VOP tem sido amplamente utilizada para fins de estratificação de risco como um fator de risco independente para mortalidade por todas as causas e por doenças cardiovasculares.^27^^,^^28^

Considerando que a aterosclerose é comumente associada ao envelhecimento arterial e doença arterial coronariana (DAC), seria de se esperar que pacientes com DAC apresentassem aumento na VOP.^29^^,^^30^ De fato, nossos resultados mostraram uma VOP pós-SVA significativamente maior em pacientes com DAC em comparação a pacientes sem DAC. No entanto, essa diferença estava ausente na VOP pré-SVA, o que sustenta a hipótese de um efeito de mascaramento pela EA, atenuando a manifestação de enrijecimento aórtico por DAC.

Estudos anteriores demonstraram disfunção vascular em pacientes obesos,^31^^,^^32^ mas, em nosso estudo, o índice de massa corporal (IMC) não foi considerado um fator preditor independente para VOP pré- e pós-operatória elevada.

O impacto estimado da EA na VOP continuará aberto para debate, pois nosso estudo teve várias limitações: 1) estudo unicêntrico; 2) natureza retrospectiva, o que leva à ausência de alguns dados (9% ausentes na análise multivariada realizada), medições de VOP pós-operatórias não programadas sistematicamente e realizadas ao mesmo tempo após a cirurgia (2 pacientes cujas cirurgias foram adiadas para 12 e 65 dias após a medição pré-operatório da VOP), e ausência de medição de longo prazo; 3) a seleção da amostra não foi aleatória nem consecutiva e seu tamanho relativamente pequeno limita a generalização externa dos resultados; 4) As medidas de VOP também apresentam limitações, como alta variabilidade de acordo com o estado do paciente; por exemplo, a pressão arterial pode não estar controlada na medição pré-operatória (todos os pacientes em jejum e com descontinuação da terapia farmacológica) em comparação com a pós-operatória (todos os pacientes sem jejum e alguns deles com pressão arterial controlada farmacologicamente).

## Conclusão

Embora alguns estudos sugiram que a rigidez aórtica é aumentada em EA devido a um componente aterosclerótico concomitante, nossos achados sugerem que EA pode atenuar a rigidez arterial real da parede aórtica, já que a gravidade de EA está inversamente relacionada à VOP, enquanto um pequeno aumento na VOP foi observado após a cirurgia de SVA e alívio do gradiente de pressão ventricular-aórtico. Como esperado, idade e PAS foram determinantes independentes de VOP mais alta em pacientes com EA grave e permaneceram os mesmos após a cirurgia. Mais estudos são necessários para fornecer uma melhor compreensão da história natural da EA e sua relação com a função vascular.
