# Supplementary material for: Alterações da Rigidez Arterial em Pacientes com Estenose Aórtica Grave Submetidos à Cirurgia de Troca Valvar
Source: Arq Bras Cardiol. 2021 Mar 3;116(3):475–82. [Article in Portuguese] doi: 10.36660/abc.20190577 (PMC8159560; doi:10.36660/abc.20190577)
Supplement: Translation

# Translation: Arterial Stiffness Changes in Severe Aortic Stenosis Patients Submitted to Valve Replacement Surgery

**Authors:** Renata Raimundo, Francisca Saraiva, Raquel Moreira, Soraia Moreira, Ana Filipa Ferreira, Rui J. Cerqueira, Mario Jorge Amorim, Paulo Pinho, António Sousa Barros, André P. Lourenço, Adelino Leite-Moreira

**Affiliations:** 1 Porto Portugal Cardiovascular Research and Development Center, Department of Surgery and Physiology, Faculty of Medicine of the University of Porto, Porto - Portugal; 2 Porto Portugal Department of Cardiothoracic Surgery, Centro Hospitalar Universitário São João, Porto - Portugal; 3 Porto Portugal Department of Anesthesiology, Centro Hospitalar Universitário São João, Porto - Portugal

**Keywords:** Aortic Valve/surgery, Aortic Valve Stenosis/surgery, Aortic Valve, Replacement /methods, Pulse Wave Analysis

## Abstract

**Background:**

Little is known about the impact of severe aortic stenosis (AS) in aortic stiffness and if there is any change after removing AS barrier with aortic valve replacement (AVR) surgery.

**Objective:**

To estimate carotid-femoral pulse wave velocity (PWV) changes after AVR surgery and to define PWV predictors in severe AS patients.

**Methods:**

Single-center retrospective cohort, including patients with severe AS who underwent AVR surgery with bioprostheses, between February 2017 and January 2019 and performed PWV measurements (Complior®) before and after the procedure (2±1 months). Before and after AVR, PWV values were compared through paired tests. The associations of PWV with clinical data were studied and linear regression models were applied to estimate pre and postoperative PWV independent predictors. The significance level was set at 5%.

**Results:**

We included 150 patients in the sample, with mean age of 72±8 years, and 51% being males. We found a statistically significant increase in PWV values after surgery (9.0±2.1 m/s vs. 9.9±2.2, p<0.001, before and after AVR, respectively) and an inverse association with AS severity variables. In the linear regression model, age and systolic blood pressure (SBP) were established as independent predictors of higher pre- and postoperative PWV, while higher mean valvular gradient emerged as a determinant of lower pre-AVR PWV.

**Conclusion:**

We documented an inverse correlation of arterial stiffness with the severity of AS in patients with AS, and a significant increase in PWV values after AVR surgery. Advanced age and higher SBP were associated with higher PWV values, although arterial function measurements were within the normal range. (Arq Bras Cardiol. 2021; 116(3):475-482)

## Introduction

Degenerative aortic stenosis (AS) is the most prevalent valvular heart disease (VHD) in developed countries and the world’s most commonly acquired VHD, being moderate or severe in 5% of patients older than 75 years in the USA.^1^ The gold standard treatment for its severe symptomatic form is Aortic valve replacement (AVR).^2^^–^^4^

The degenerative process of the aortic valve (AV), which results in AS, has pathophysiological changes that are similar to the atherosclerotic process responsible for increased arterial stiffness.^5^ The scientific community, therefore, is led to postulate that, in AS, some degree of vascular dysfunction and consequently arterial stiffness could also be present.^6^

Aging also contributes to vascular stiffening, increasing aortic pressure. Aortic pulse wave velocity (PWV) is the gold standard, non-invasive, and most reproducible method to assess arterial stiffness.^7^ Essentially, it evaluates aortic elastic recoil capacity, which is diminished in a stiffer aorta, translating into a higher PWV value.^8^^–^^10^ So, in an AS patient, we expect to find a higher preoperative PWV, that may be recovered after AVR. However, some studies have shown that this association may not be linear,^11^ meaning that even after the procedure that reliefs valvular obstruction, a high PWV measurement may be seen (or even higher than the pre-intervention assessment), representing an increase in vascular load.^12^

There is a strong association between a higher PWV and systolic hypertension, as well as other cardiovascular (CV) risk factors and atherosclerotic disease.^13^^,^^14^ PWV has also been suggested to predict fatal and nonfatal CV events, such as stroke or aortic and coronary syndromes.^8^^,^^9^^,^^15^^,^^16^

There is conflicting evidence regarding arterial function changes after AVR, and whether PWV can be a marker of AS severity or not. In this setting, this study aimed to clarify this association.

## Objective

The main goal of this study was to evaluate arterial stiffness before and after AVR surgery in patients with severe AS, using a PWV measurement equipment. We also aimed to identify the predictors of pre and postoperative PWV results in these patients.

## Methods

### Study Design and Patients

Single-center retrospective study, including 150 patients with severe AS who underwent AVR surgery with bioprostheses between February 2017 and January 2019, with pre and postoperative PWV measurements. Patients with concomitant moderate or severe aortic regurgitation or multiple procedures were excluded.

### Data Collection and Variables

Preoperative, surgical, and postoperative data were collected from medical records and databases. Regarding preoperative variables, besides PWV values, we also collected data on blood pressure, demographic information, cardiovascular risk factors, ongoing medical therapy, functional status, symptoms, and transthoracic echocardiogram. Cross-clamp (XCT) and cardiopulmonary bypass time (CPBT), aortic valve disease etiology, and type of prosthesis were the main surgical variables. Follow-up variables were the results of transthoracic echocardiogram (mean at 3.9±1.6 months of follow-up) and PWV evaluation.

The local ethics committee approved this study, and all data were anonymized for analysis.

### PWV Measurement

A noninvasive method (Complior^®^ Analyse) was used to evaluate arterial stiffness through carotid-femoral PWV before and after AVR surgery. After a few minutes in supine resting position to stabilize heart rate and blood pressure, the blood pressure was assessed using a standard sphygmomanometer. Carotid to femoral distance was measured using a metric tape, and data were input in a specific software. Femoral and carotid sensors were held until the software reached stabilized lines and the best quality of signal (above 90%). Each patient was submitted to at least two PWV measurements in each session. These measurements were done at patients’ admission to the Cardiothoracic Surgery Department on the day before or on the same day of the surgery. The postoperative evaluation occurred on average 2.2±1.4 months after surgery.

### Statistical Analysis

The distribution of continuous data was verified through visual analysis of the histograms and confirmed by Shapiro Wilk test. Continuous variables are presented as mean and standard deviation. Categorical variables are depicted in absolute and relative frequencies. Paired samples *t* test was used to compare continuous variables at two distinct moments. Correlations between PWV and other continuous variables were assessed through Pearson correlation test. PWV values were compared between groups using independent *t* test. A multivariable linear regression model was built to estimate PWV predictors based on clinical variables relevant to arterial stiffness evaluation. The regression’s assumptions were checked, residuals were normally distributed and the independent variables were not highly correlated. A significance level of 5% was used. Statistical analyses were performed using the Statistical Package for the Social Sciences (SPSS) version 24 and the R language environment version 3.6 (R Core Team - 2018. R: A language and environment for statistical computing. R Foundation for Statistical Computing, Vienna, Austria. URL https://www.R-project.org/. Frank E Harrell Jr - 2019. rms: Regression Modeling Strategies. R package version 5.1-3.1).

## Results

### Sample

Sample characterization is depicted in Table 1. Mean age of subjects was 72±8 years, and 51% of them were males. Arterial hypertension was present in 125 patients (83%), dyslipidemia in 114 (76%), diabetes in 52 (35%), and history of smoking in 36 (24%). Forty (27%) patients were admitted with New York Heart Association (NYHA) functional class ≥III. Most patients (91%) were on anti-hypertensive drugs at admission.

**Table 1:** Baseline characteristics

Variable	n=150
Age, in years, mean (SD)	72.5 (7.6)
Male sex, n (%)	77 (51.3)
NYHA ≥ III, n (%)	40> (26.9)
Hypertension, n (%)	125 (83.3)
Currently on anti-hypertensive drugs (%)	136 (90.7)
Diabetes, n (%)	52 (34.9)
Dyslipidemia, n (%)	114 (76.0>)
History of smoking, n (%)	36 (24.0)
Body mass index, kg/m^2^, mean (SD)	28.6 (4.3)
Obesity (BMI ≥30.00 kg/m²), n (%)	55 (36.7)
Coronary artery disease, n (%)	19 (12.7)
Extracardiac arteriopathy, n (%)	22 (14.7)
Chronic kidney disease (CC <85ml/min), n (%)	87 (58.0)
Creatinine Clearance (CC), ml/min, mean (SD)	83.7 (29.7)

BMI: body mass index; min: minute; NYHA: New York Heart Association; SD: standard deviation. Extracardiac arteriopathy was considered if the patient had claudication, carotid occlusion or >50% stenosis, amputation due to arterial disease, past or planned intervention on the abdominal aorta, limb arteries or carotids, or history of stroke. Coronary artery disease was defined when patients had undergone percutaneous coronary intervention in the past, or coronary stenosis >50%, but without indication for surgery.

During surgery, 12% of patients were confirmed for congenital etiology (Table 2).

**Table 2:** Perioperative data

Variable	n=150
**Aortic valve etiology, n (%)**	
Degenerative	132 (88.0)
Congenital	18 (12.0)
CPB time, minutes, mean (SD)	78 (26)
Aortic clamp time, minutes, mean (SD)	57 (20)

CPB: cardiopulmonary bypass; SD: standard deviation.

### Pre and Postoperative PWV

A significant postoperative increase in PWV values was observed, ranging from 9.0±2.1 m/s to 9.9±2.2 m/s after AVR surgery (p<0.001, Figure 1A).

**Figure 1:** Violin plots for pre- (red) and post- (blue) operative values of pulse wave velocity (A), systolic blood pressure (B), aortic valve mean gradient (C) and aortic valve area (D).

### Follow-up Data

Systolic blood pressure (SBP), mean aortic valve gradient (MVG) and aortic valve area (AVA) values before and after AVR surgery are represented in Figure 1 (Panels B, C and D, respectively).

### PWV Associations

Figure 2 depicts the univariate analysis considering PWV associations with potential predictors. Preoperative and postoperative PWV had positive correlations with age, SBP and mean blood pressure (MBP), but inversely associated with aortic stenosis severity variables. We found no differences in PWV according to gender, arterial hypertension, diabetes, smoking, or bicuspid AV subgroups.

**Figure 2:** Scatter plots for pre- (blue) and post- (red) operative PWV relationships with age, SBP: systolic blood pressure, mean pressure, BMI: body mass index, clearance creatinine, AVA: aortic valve area, stroke volume, diastolic pressure, ejection fraction, aortic mean valve gradient and aortic valve maximum gradient.

In the multivariable linear regression model, age and SBP were independent predictors of higher preoperative PWV, while higher MVG was a predictor of lower preoperative PWV (Table 3 and Figure 3). Age and SBP were considered independent predictors of higher postoperative PWV (Table 4 and Figure 4).

**Table 3:** Summary of the multivariable regression analysis (dependent variable: preoperative PWV)

Variable	β	SE	b	*p*
Intercept	0.593	2.576	0.23	0.818
Systolic blood pressure	0.020	0.008	2.42	0.017
Diabetes>	0.428	0.344	1.24	0.215
Clearance creatinine	-0.001	0.007	-0.14	0.889
Coronary artery disease>	0.531	0.498	1.07	0.288
BMI	0.059	0.040	1.46	0.147
Age	0.070	0.025	2.87	0.004
Sex	0.244	0.336	0.73	0.469
MVG	-0.029	0.011	-2.75	0.007
Bicuspid aortic valve	-0.299	0.498	-0.60	0.549

BMI: body mass index; β: unstandardized regression coefficient; b: standardized coefficient; MVG: mean valve gradient; SE: standard error of the coefficient.

**Figure 3:** Multivariable linear regression model of preoperative PWV and estimated versus actual values. BAV: bicuspid aortic valve; BMI: Body mass; CAD: coronary artery disease; MVG: mean valve gradient; SBP: systolic blood pressure.

**Table 4:** Summary of the multivariable regression analysis (dependent variable: postoperative PWV)

Variable	β	SE	b	p
Intercept	-2.706	2.391	-1.13	0.260
Systolic blood pressure	0.037	0.009	4.11	<0.001
Diabetes	0.448	0.352	1.27	0.205
Coronary artery disease>	0.837	0.508	1.65	0.102
BMI	0.023	0.040	0.58	0.564
Age	0.097	0.023	4.13	<0.001
Sex	0.287	0.351	0.82	0.415
MVG	-0.056	0.038	-1.46	0.146

BMI: body mass index; β: unstandardized regression coefficient; b: standardized coefficient; MVG: mean valve gradient; SE: standard error of the coefficient.

**Figure 4:** Multivariable linear regression model of postoperative PWV and estimated versus actual values. BMI: body mass index; CAD: coronary artery disease; MVG: mean valve gradient; SBP: systolic blood pressure.

## Discussion

This retrospective study showed an inverse correlation of arterial stiffness with severity of AS and a significant increase in PWV values after AVR surgery in patients with severe AS. Advanced age and higher SBP were associated with higher PWV values, although arterial function measurements were within the normal range.

PWV reflects arterial stiffening due to loss of aortic functional elastic properties. Arterial stiffness is one of the first manifestations of reversible structural damage to the vessel wall, and PWV is considered a technique with clinical applicability both to identify and stratify cardiovascular disease.^8^^,^^16^

Assessing the impact of degenerative AS on the arterial tree remains a challenge, as the mechanisms underlying the interaction of vascular and valvular function remain mostly unknown.^17^

Liu et al.^5^ showed the association of increased PWV with higher calcium score in the AV.^5^ These results suggest that calcium deposition is an important pathway in the degenerative process of the aortic valve and wall. In addition, they showed an association between increased PWV and AV pressure gradient assessed by echocardiography. Similar pathophysiological changes are probably shared between AV degeneration and large arteries stiffening. Korkmaz et al.^18^ estimated arterial stiffening using the cardio-ankle vascular index and found higher arterial stiffness in patients with aortic valve sclerosis,^18^ suggesting that AV degeneration and large arteries stiffening could share similar pathophysiological changes. Indeed, Emir Cantuk et al.^11^ reported a significant association between AS severity and increased PWV.^11^ Contrarily, our study shows an inverse correlation between AV gradients and preoperative PWV. This might partially be explained by the upstream obstruction that may influence measurements of arterial properties, masking the real effects of vascular load on the aorta. El-Chilali et al.,^6^ who studied older patients (>70 years old) with severe AS and measured PWV invasively, observed the same: an inverse correlation between MVG and PWV.^6^ This hypothesis is strengthened by the increase in PWV that we found after AVR surgery.

Only a limited number of studies have evaluated aortic vascular function in patients with severe AS after intervention so far.^11^^,^^19^^,^^20^ Canturk et al.^11^ did not find significant differences in PWV after surgery.^11^ On the other hand, Nemes et al.^20^ demonstrated a vascular function improvement one year after AVR.^20^ In our study, PWV increased significantly after AVR surgery. Some mechanisms are considered to possibly explain this result: the relief of valvular obstruction after AVR leads the arterial tree to operate at a higher-pressure level, increasing the vascular load. In fact, Yotti et al.^19^ showed that the relief of the outflow obstruction immediately raises arterial pressures and vascular impedance, inducing a stiffer vascular behavior^19^. Overall, these findings are reflected in clinical practice, since it is common to initiate anti-hypertensive drugs after AS correction, either by transcatheter aortic valve implantation (TAVI) or surgery.^19^^,^^21^^,^^22^ Another explanation, stated by Barbetseas,^12^ suggests that the increase in arterial stiffness after AVR surgery occurs due to aortic wall injury and *vasa vasorum* destruction, as well as aortic wall fiber composition alteration, resulting in aortic stiffening. However, we should reinforce that this decrease in aortic distensibility was evaluated one week after AVR, whereas six months after AVR, the aortic function improved, reaching levels similar to preoperative ones.^12^ It may represent a transient effect described by the authors as “aortic root stunning”.^23^

A study that compared TAVI and AVR showed significant changes in PWV only in surgical patients, suggesting that, in TAVI patients, the elastic properties are maintained because there is no surgical manipulation.^24^

In our study, PWV was also found to be significantly associated with age, which was considered an independent predictor of higher PWV values. PWV alteration in the elderly is already well established in the literature.^8^^,^^17^^,^^25^ Studies have shown an increase in PWV with aging probably related to aortic dilation and stiffening, since impedance increases and arterial compliance decreases with aging.^8^^,^^26^ Our study supports these results, with age proven to be strongly correlated with both pre- and postoperative PWV.

Systemic hypertension was also shown to be a predictor of increased PWV,^9^^,^^27^ which is in line with our results as higher SBP was associated with higher PWV. Since over 80% of our sample had systemic hypertension, we did not find substantial differences between patients with and without this risk factor, but we recognize that PWV has been widely used for risk stratification purposes as an independent risk factor for all-cause and CV mortality.^27^^,^^28^

Considering that atherosclerosis is commonly associated with arterial aging and coronary artery disease (CAD), it would be expected that patients with CAD had increased PWV.^29^^,^^30^ In fact, our results showed a significantly higher post-AVR PWV in patients with CAD in comparison with non-CAD patients. However, this difference was absent in pre-AVR PWV, which supports the hypothesis of a masking effect in the presence of AS, blunting the manifestation of aortic stiffening induced by CAD.

Previous studies have shown vascular dysfunction in obese patients,^31^^,^^32^ but in our study, body mass index (BMI) was not considered an independent predictor factor for higher pre- and postoperative PWV.

The estimated impact of AS in PWV will still be open to debate, as our study had several limitations: 1) single-center study; 2) retrospective nature, which leads to some missing data (9% missing in the multivariable analysis performed), postoperative PWV measurements not systematically programmed and performed at the same time after surgery (2 patients whose surgeries were postponed to 12 and 65 days after preoperative PWV measurement), and absence of long-term measurement; 3) the sample selection was neither randomized nor consecutive, and its relatively small size limits the external generalization of results; 4) PWV measurements also have limitations such as high variability according to patient status; for example, blood pressure could be not controlled in the preoperative measure (all patients fasting and with discontinuation of pharmacological therapy) compared to postoperative measure (all patients without fasting and some of them with pharmacologically-controlled blood pressure).

## Conclusion

Although some studies suggest that aortic stiffness is increased in AS due to a concomitant atherosclerotic component, our findings suggest that AS may blunt the real arterial stiffness of the aortic wall, as the severity of AS is inversely related to PWV, while a small increase in PWV was observed after AVR surgery and ventricular-aortic pressure gradient relief. As expected, age and SBP were independent determinants of higher PWV in patients with severe AS and remained the same after surgery. Further studies will be needed to provide better insight into the natural history of AS and its relation to vascular function.
